# Case Report: Respiratory paralysis associated with polymyxin B therapy

**DOI:** 10.3389/fphar.2022.963140

**Published:** 2022-08-29

**Authors:** Yachan Ning, Yanqi Chu, You Wu, Ying Huang, Chunmei Wang, Li Jiang

**Affiliations:** ^1^ Department of Intensive Care Medicine, Xuanwu Hospital, Capital Medical University, Beijing, China; ^2^ Department of Pharmacy, Xuanwu Hospital, Capital Medical University, Beijing, China; ^3^ Department of Urology Surgery, Xuanwu Hospital, Capital Medical University, Beijing, China

**Keywords:** respiratory paralysis, neurotoxicity, neuromuscular blockage, polymyxin B, colistin

## Abstract

Polymyxin B (PMB) and colistin are bactericidal polypeptide antibiotics discovered in 1947 and 1949 for the treatment of gram-negative bacterial infections. Polymyxin was used clinically in the 1950s, but it was gradually replaced by other antibiotics in the 1980s because of its high nephrotoxicity and neurotoxicity. In recent years, the increase of multidrug-resistant negative bacteria has led to the resurgence of polymyxin use. However, its side effects are not clear. Respiratory paralysis caused by PMB-related neuromuscular blockade is a rare but potentially fatal effect. We report a case of respiratory paralysis probably caused by polymyxin B infusion.

## Introduction

Polymyxin B and colistin have a bactericidal effect on the cell wall of gram-negative organisms, resulting in the change in plasma membrane permeability and cell death (Myint et al., 2016). Due to the significant nephrotoxicity and neurotoxicity, their use was significantly reduced from the 1970s to the 2000s (Falagas and Kasiakou, 2006). Their clinical application has recently revived and played an important role in treating the untreated gram-negative infection (Tsuji et al., 2019). In the past 2 years, the number of patients using polymyxin B in our hospital has significantly increased; we report one patient who experienced acute respiratory distress that was probably induced by polymyxin infusion.

## Case presentation

The patient was a 43-year-old man with a history of primary hypertension and chronic renal failure who underwent renal transplantation in September 2021. He had no family and psychosocial history. Informed consent of the patient was obtained. After the operation, the patient received routine antirejection therapy but still needed routine renal replacement therapy due to delayed graft function (DGF). He was admitted to the intensive care unit (ICU) 5 months after the operation with hypoxemia caused by cytomegalovirus pneumonia and pneumocystis jirovecii pneumonia. After antiviral (ganciclovir 0.25 g qd po) and antifungal (caspofungin 50 mg qd ivgtt, trimethoprim-sulfamethoxazole 0.96 g tid po) treatment, hypoxemia improved significantly. Therefore, caspofungin was stopped after 3 weeks, and ganciclovir and trimethoprim-sulfamethoxazole were continued. However, he then developed bloodstream infections presenting with chills and high fever caused by carbapenem-resistant *Enterobacter cloacae*, which were sensitive only to tigecycline [minimum inhibitory concentration (MIC) = 1 mg/L]. He was treated with meropenem (0.5 g q12 h ivgtt) and tigecycline (100 mg q12 h ivgtt) before culture was reported but his condition did not improve. Then, meropenem was changed to PMB (50 mg q12 h ivgtt). His bloodstream infection was controlled, and he was returned to the general ward to continue the current treatment. PMB was injected intravenously at a dose of 50 mg every 12 h. Before infusion of PMB, the patient rested comfortably on oxygen through a nasal cannula (on a FiO_2_ of 30%) with a recorded blood oxygen saturation (SaO_2_) of 100% and a respiratory rate of 16–20 breaths/min. His serum creatinine level was 5.63 mg/dl before the first dose of PMB. An hour after the 7th injection of PMB, he developed acute dyspnea with decreasing SaO_2_, followed by a rapid loss of consciousness. He received emergency endotracheal intubation and soon became conscious. An arterial blood gas sample taken shortly after intubation showed hypercapnia (pH 7.165, PCO2 66.1, and PaO_2_ 160 on 100% FiO_2_). He did not need any vasopressor support during respiratory failure and was extubated 12 h later. After extubation, the patient complained of laborious breathing and was provided a noninvasive ventilator. Two hours after the 11th PMB infusion, he developed respiratory arrest and transient loss of consciousness. The blood gas sample extracted immediately showed that it was still hypercapnia (pH 6.936, PCO_2_ too high to be measured, and PaO_2_ 94.8 on 21% FiO_2_). The patient underwent endotracheal intubation again and was readmitted to the ICU. After each crisis, carotid ultrasound, transcranial doppler, transthoracic echocardiogram, vocal cord, and supraglottic area examination were performed, which were normal. The Naranjo adverse drug reaction probability scale of the patient was 9, which showed a definite relationship between PMB infusion and respiratory failure (Table 1). The score of other drugs was less than 0. Therefore, polymyxin-induced neuromuscular blockade was suspected, and PMB was discontinued immediately. After discontinuation of PMB, his serum creatinine level was 5.73 mg/dl. Three days later, he was extubated without any recurrence of respiratory failure or respiratory arrest. The blood gas sample after discontinuation of PMB showed that it was significantly improved (pH 7.399, PCO_2_ 41, and PaO_2_ 97.4 on 29% FiO_2_). The patient later recalled that he was fully aware of the crisis but felt unable to breathe. He was discharged 1 week after extubation without any recurrence of respiratory distress. At present, the patient has been discharged for 3 months and is in a stable condition.

**TABLE 1:** ADR probability scale.

To assess the adverse drug reaction, please answer the following questionnaire and give the pertinent score				
	Yes	No	Do not know	Score
1. Are there previous conclusive reports on this reaction?	+1	0	0	+1
2. Did the adverse event appear after the suspected drug was administered?	+2	−1	0	+2
3. Did the adverse reaction improve when the drug was discontinued or a specific antagonist was administered?	+1	0	0	+1
4. Did the adverse reaction reappear when the drug was readministered?	+2	−1	0	+2
5. Are there alternative causes (other than the drug) that could on their own have caused the reaction?	−1	+2	0	+2
6. Did the reaction reappear when a placebo was given?	−1	+1	0	+1
7. Was the drug detected in the blood (or other fluids) in concentrations known to be toxic?	+1	0	0	0
8. Was the reaction more severe when the dose was increased or less severe when the dose was decreased?	+1	0	0	0
9. Did the patient have a similar reaction to the same or similar drugs in any previous exposure?	+1	0	0	0
10. Was the adverse event confirmed by any objective evidence?	+1	0	0	0
				Total score 9

ADR, adverse drug reaction.

ADR was assigned to a probability category from the total score as follows: definite ≥9, probable 5– 8, possible 1–4, and doubtful ≤0.

## Discussion

The purpose of our case study is to describe respiratory paralysis due to the neurological toxicity associated with PMB. The incidence of polymyxin-related neurotoxicity reported in the literature is 0%–7% (Landman et al., 2008). Respiratory muscle paralysis is a rare but potentially fatal complication of polymyxin. In the 1950s and 1960s, cases of respiratory paralysis caused by neuromuscular blockage associated with PMB or colistin have been reported in the literature (Fekerty et al., 1962; Pohlmann, 1966; Lindesmith et al., 1968). In the past 30 years, very few studies have described respiratory paralysis caused by polymyxin B. Our patient underwent respiratory failure twice of unknown etiology, required intubation and mechanical ventilation after PMB injection, and suffered respiratory muscle weakness, which was consistent with the neurotoxicity of PMB.

The neurological toxicity manifests as dizziness, muscle weakness, facial and peripheral paresthesia, visual disturbances, vertigo, confusion, hallucinations, seizures, partial deafness, ataxia, and neuromuscular blockade (Falagas and Kasiakou, 2006). The mechanism of neuromuscular blockade was that polymyxin may inhibit the action of acetylcholine at the neuromuscular junction, prolong depolarization, consume calcium, and induce histamine release (Lindesmith et al., 1968). The possibility of neurotoxicity is directly related to the concentration of the polymyxin active form in the blood (Hoeprich, 1970).

Previous studies showed that the main risk factor for the polymyxin-associated neuromuscular block was renal disease (Lindesmith et al., 1968; Wunsch et al., 2012). This may be due to the accumulation of polymyxin caused by renal insufficiency, which leads to neurotoxicity. However, polymyxin itself can also cause or aggravate renal function injury (Mendes et al., 2009). In this case, the patient had severe renal insufficiency and developed unexplained respiratory failure. However, no PMB plasma levels were extracted and analyzed, and we cannot confirm that the patient did not clear the drug due to his renal impairment. Naranjo et al. first proposed the Naranjo adverse drug reaction probability scale for adverse drug reactions (ADRs) in the 1980s, which was widely used after improvement and perfection (Naranjo et al., 1981; Naranjo et al., 1992). The scale is composed of 10 medical problems related to ADRs with predetermined scores (Table 1). It is mainly used to evaluate and determine the correlation and tightness between drug use and ADR. The Naranjo adverse drug reaction probability scale was 9, which indicated a definite relationship between the administration of PMB and respiratory failure in the patient. The patient developed respiratory paralysis again after polymyxin treatment and recovered rapidly after stopping the drug, which increased certainty. Risk factors that may potentially trigger the development of neurotoxicity include the combination of polymyxin with muscle relaxants, anesthetics, sedatives, narcotic drugs, or corticosteroids (Falagas and Kasiakou, 2006). He received immunosuppressors, hormones, and other antibiotics after renal transplantation which might have potentially increased the neuromuscular block.

Lindesmith described 21 cases of reversible respiratory paralysis associated with polymyxin therapy, including six cases that used PMB (Lindesmith et al., 1968). One of the six cases died. The mortality of respiratory paralysis related to the use of polymyxins has not been reported in the literature. The treatment of polymyxin-related neurotoxicity is usually supportive including controlled ventilation and discontinuance of polymyxin. Due to rapid recovery, endotracheal intubation is usually given instead of tracheotomy. Calcium injection is inconclusive in reversing neurotoxicity (Lindesmith et al., 1968; Hoeprich, 1970). The limitations of this study were a lack of PMB plasma levels and electromyography (EMG).

## Conclusion

This case illustrates the importance of recognizing respiratory paralysis associated with polymyxin B. The awareness of polymyxin B’s potentially fatal effects must be kept in mind when using this antibiotic, especially in patients with renal insufficiency.

## Data Availability

The original contributions presented in the study are included in the article/supplementary material; further inquiries can be directed to the corresponding authors.
